# Cancer among syrian refugees living in Konya Province, Turkey

**DOI:** 10.1186/s13031-022-00434-4

**Published:** 2022-01-31

**Authors:** Tezer Kutluk, Mehmet Koç, İrem Öner, İbrahim Babalıoğlu, Meral Kirazlı, Sinem Aydın, Fahad Ahmed, Yavuz Köksal, Hüseyin Tokgöz, Mustafa Duran, Richard Sullivan

**Affiliations:** 1 Department of Pediatric Oncology, Hacettepe University Faculty of Medicine and Cancer Institute, 06100 Ankara, Turkey; 2 Provincial Directorate of Health Konya, Konya, Turkey; 3 Medical Oncology Unit, Ministry of Health City Hospital, Konya, Turkey; 4 Radiation Oncology Unit, Ministry of Health City Hospital, Konya, Turkey; 5 Department of Pediatric Oncology, Selçuk University Meram Faculty of Medicine, Konya, Turkey; 6 Department of Pediatric Hematology, Necmettin Erbakan University Faculty of Medicine, Konya, Turkey; 7 Hematology Unit, Ministry of Health City Hospital, Konya, Turkey; 8 Institute of Cancer Policy, Conflict and Health Research Group, King’s College London, London, UK

**Keywords:** Cancer, Syrian refugees

## Abstract

**Background:**

With more than 3.6 million Syrian refugees Turkey hosts the world's largest number of Syrians. Considering the morbidity, mortality, and healthcare spending, cancer is one of the leading health and economic burden for patients and healthcare systems. However, very limited information available in the scientific literature to understand the burden and characteristics of cancer in countries hosting Syrian refugees. The aim of the present study is to evaluate the demographic and clinical characteristics, treatment outcome of Syrian cancer patients living in Konya, Turkey.

**Methods:**

We retrospectively reviewed medical records of Syrian cancer patients at three major institutions from 2005 to 2020. The information regarding demographic and clinical characteristics of patients were identified. The number of days between the first symptom and diagnosis was considered as the “diagnostic interval”. Patients who failed to attend clinics within four weeks of appointment were assumed abandoned treatment. Survival curves were estimated using the Kaplan–Meier method.

**Results:**

We identified 230 adult and 38 children refugee diagnosed with cancer during the study period. With regards to adult patients, there were 114 (49.6%) male and 116 (50.4%) female. The median age at diagnosis was 52.4, 47.3 years for male, female respectively. The five most common cancer by site among all were; breast (24.8%), colorectal (10.9%), lung (7.4%), central nervous system (CNS) (7.0%), and stomach (5.2%). 93 (40.4%) had metastatic disease at diagnosis. The overall survival probability was 37.5% at five years for the adult population. Data were extracted for 20 boys and 18 girls with childhood cancer. Their median age at diagnosis was 5.8 and 6.0 years respectively. The three most common childhood cancer were; leukemias (21.1%), lymphomas (21.1%), and CNS (13.2%). Excluding leukemia, 13 (43.3%) of childhood cancer cases had the advanced disease at diagnosis. Three year survival probality was 69.5%. The median diagnostic interval for adult and childhood cancer was 96.5 (IQR = 53–165) and 23 (IQR = 13.5–59) days respectively. Twenty-one adults and four children had treatment abandonment.

**Conclusion:**

This study contributes to understanding the burden of cancer among Syrian refugees living in Konya, growing health issue for refugees. Larger and prospective studies will help to measure the real burden and compare the difference in cancer risk factors, care, and outcomes among the refugee and host populations.

**Supplementary Information:**

The online version contains supplementary material available at 10.1186/s13031-022-00434-4.

## Introduction

The Syrian conflict, now in its 11th year, is one of the worst humanitarian crisis of the twenty-first century. The most recent data suggest that 6.7 million people are displaced inside Syria and 5.6 million people have been forced to live in other countries including the bordering countries [1]. Turkey shares a 911-km long border with Syria and has granted temporary protection inside Turkey to more than 3.6 million Syrians [2]. The most urgent health problems of newly arrived refugees were physical trauma (accidental injuries, burns, gunshot and blast injuries etc.), infection, malnutrition, and mental health. However, over time the humanitarian crisis had to adapt to managing a wide ragne of NCDs (non-communicable diseases), including cancer becoming a major part of healthcare needs [3–6].

The right to health is a fundamental part of the Turkish constitution [7]. The refugees’ access to health care is a priority issue in Turkey. Since the start of the refugee crisis all registered Syrians have been able to access the primary and secondary health services. Moreover, to improve access Migration Health Centers and mobile health units were also established. The Turkish government also started a scheme to cover the cost of refugee’s health care with the aim to give access to Syrian refugees to a wide range of health services including cancer care.

Cancer is one of the leading causes of morbidity and mortality in almost all populations around the world. Cancer represents a significant burden of disease in Syria. Over the course of the conflict it has still been a leading cause of death in Syria after heart disease and injuries [8]. Since the start of the conflict Syrian cancer patients have not been able to access timely cancer care, either in Syria nor in several countries hosting them [5]. Turkey has been the exception. However, the awareness and utilization of cancer services, including screening has remained very low amongst Syrian refugees in Turkey [9]. In addition providing such cancer services has been a substantial economic issues for both refugees and host country [10]. Despite nearly a decade of experience there are few studies that have quantified refugee cancer care needs that can inform policy-makers in hosting countries and the wider international humanitarian community. The present study aimed to evaluate the demographic, clinical characteristics and treatment outcomes of Syrian cancer patients living in the Konya province of Turkey.

## Methods

### Data sources

This was a retrospective study of all cancer patients registered between 1 January 2005 and 31 December 2020 at Selçuk and Necmettin Erbakan University Hospitals and Ministry of Health City Hospital of Konya who were designated on records as Syrian nationals under temporary protection. Konya is the largest province of Turkey by size and currently hosts 121,064 Syrian refugees. The number of refugees who reside in Konya has increased markedly in previous years, now ranking 10th among the cities with highest number of refugee population [11]. Data collection was approved by the Konya Health Directorate Ethical Review Board. Data from hospital records were extracted by the trained physicians using the registered ID number given to Syrian refugees by the Turkish Directorate General of Migration Management, as well as the International Classification of Diseases, tenth edition (ICD-10) [12], and International Classification of Childhood Cancer third edition (ICCC-3) [13] codes at the admission and/or consultation of confirmed cases of cancer. Data of the patients who were diagnosed at other centers and applied to start or continued their treatment in the above hospitals were also included. The study sample did not include illegal immigrants or medical tourists.

### Variable selection

The variables extracted for analysis included: date of birth, sex, place of residence, comorbidities, smoking status, date of first cancer-related symptom, the method used to detect cancer, date of cancer diagnosis, place of diagnosis, disease status at first presentation, ICD-10 topography and morphology codes for adult cancer site, ICCC-3 code for childhood cancer site, Surveillance Epidemiology and End Results (SEER) classification of cancer stage [14], treatment modalities, place of residence during the treatment, date of last hospital visit, the status of the patient at last visit, and date of death. The diagnostic interval was defined as the number of days between the date of the first cancer symptom and the date of diagnosis. Relapse was defined as new evidence of cancer after attaining remission. Treatment abandonment was defined as failure to attend the clinic within four weeks of a prescribed appointment throughout treatment, except in situations when treatment is contraindicated for medical reasons. We noted missing data on some variables and consider them missing random.

### Statistical analysis

Data were analyzed using the SPSS version 21. Categorical variables were presented as frequency and percentage whereas continuous variables were presented as mean and median. Survival curves were estimated according to the Kaplan–Meier method and *p* value of < 0.05 was considered statistically significant.

## Results

### Cancer among adults

Records of 230 adult cancer patients (older than 16 years) were identified and extracted for analysis. Among them 226 (98.3%) patients had their first diagnosis between September 2011 and December 2020 (Additional file 1: Table 1). There were 114 (49.6%) male and 116 (50.4%) female patients in the cohort. The median age at diagnosis was 52.4 years for male (range 17–82 years) and 47.3 years for female patients (range 17–94). 77.8% (n = 179) had their first diagnosis in Turkey compared to 21.7% (n = 50) who were first diagnosed in Syria and one had missing information.

A total of 206 (89.6%) patients lived in Turkey during their cancer treatment, whereas seven patients were visiting Turkey just for treatment and returning back to Syria while holding refugee status (Visiting Refugee). The cases with missing information were shown in Table 1. Smoking data were available for only 160 patients; 49 (30.6%) of them were smokers. Smoking rates were higher in male compared to female (68.6% vs. 3.2%) population among the 160 patients for whom smoking data was available (Table 1) The median duration of smoking was 30 years (range 3–50 years), among them 40 (81.6%) smoked one pack per day, seven patients (14.3%) smoked more than one pack per day, and two pateints had missing data. Co-morbidity was found for 47 patients (20.4%); hypertension, diabetes mellitus and ischemic heart disease were the most common diseases (Additional file 1: Table 2).

**Table 1:** Characteristics of Syrian refugee patients

Characteristics	Adult						Children					
	Male		Female		Total		Male		Female		Total	
	Median	Range	Median	Range	Median	Range	Median	Range	Median	Range	Median	Range
Age (years)	52.4	16.7–81.8	47.3	17.0–94.0	49.0	16.7–94.0	5.78	0.2–13.1	5.97	0.6–15.6	5.81	0.2–15.6
	n	%	n	%	n	%	n	%	n	%	n	%
*Country of first diagnosis*												
Turkey	97	85.1	82	70.7	179	77.8	19	95.0	17	94.4	36	94.7
Syria	16	14.0	34	29.3	50	21.7	1	5.0	1	5.6	2	5.3
Other*	1	0.9	–	–	1	0.4	–	–	–	–	–	–
*Country of residence during the last three years*												
Turkey	76	66.7	70	60.3	146	63.5	19	95.0	13	72.2	32	84.2
Syria	20	17.5	21	18.1	41	17.8	–	–	5	27.8	5	13.2
Missing data	18	15.8	25	21.6	43	18.7	1	5.0	–	–	1	2.6
*Country of residence during treatment*												
Turkey	104	91.2	102	87.9	206	89.6	19	95.0	15	83.3	34	89.5
Syria*	4	3.5	3	2.6	7	3.0	–	–	3	16.7	3	7.9
Missing data	6	5.3	11	9.5	17	7.4	1	5.0	–	–	1	2.6
*Smoking*												
Yes	46	40.4	3	2.6	49	21.3	N/A	N/A	N/A	N/A	N/A	N/A
No	21	18.4	90	77.6	111	48.3	N/A	N/A	N/A	N/A	N/A	N/A
Missing data	47	41.2	23	19.8	70	30.4	N/A	N/A	N/A	N/A	N/A	N/A
Total	114	100.0	116	100.0	230	100.0	20	100.0	18	100.0	38	100.0

*N/A* non-applicable

*Visiting Turkey just for treatment and returning back to Syria while holding refugee status (Visiting Refugee)

When the whole group was examined, 82.2% (n = 189) were newly diagnosed whereas 11.3% (n = 26) presented with cancer recurrence, and 6.5% (n = 15) of patients had been diagnosed in other centers and applied to continue their treatment in the hospitals in Konya during the period of this study. Cancer was diagnosed by histopathological examination in 97.4% (n = 224) patients. When the stages of patients at the time of diagnosis were examined 19.1% (n = 44) patients had the local disease (SEER 0 and 1), 40% (n = 92) had the loco-regional disease (SEER 2–5) and, 40.4% (n = 93) had metastatic diseases (SEER 7). The abandonment rates were estimated at 9.2% (n = 21) for this cohort and were higher in male than female patients (11.5% vs. 6.9%) (Table 2). The reasons for abandonment were unknown in 12 cases, never started in eight cases, one interrupted during the migration/social reasons (Table 2).

**Table 2:** Tumor characteristics in adult Syrian refugee patients

Characteristics		Male		Female		Total	
		n	%	n	%	n	%
Disease status	Newly diagnosed disease	98	86.0	91	78.4	189	82.2
	Relapsed disease	13	11.4	13	11.2	26	11.3
	Others	3	2.6	12	10.3	15	6.5
Diagnostic method	Histological diagnosis from primary	103	90.4	110	94.8	213	92.6
	Histological diagnosis from metastasis	7	6.1	4	3.4	11	4.8
	Cytological diagnosis	2	1.8	–	–	2	0.9
	Clinical examination	1	0.9	2	1.7	3	1.3
	Unknown	1	0.9	–	–	1	0.4
Dissemination of the disease (adults)	Local (SEER 0–1)	26	22.8	18	15.5	44	19.1
	Regional (SEER 2–5)	39	34.2	53	45.7	92	40.0
	Metastatic (SEER 7)	49	43.0	44	37.9	93	40.4
	Unknown (SEER 9)	–	–	1	0.9	1	0.4
Treatment abandonment	Yes	13	11.5	8	6.9	21	9.2
	No	101	88.5	108	93.1	208	90.8
Total		114	100.0	116	100.0	230	100.0

The five most common cancer site specific cancers were; breast (n = 57, 24.8%), colorectal (n = 25, 10.9%), lung and bronchus (n = 17, 7.4%), central nervous system (n = 16,7.0%), and stomach (n = 12, 5.2%). The five most common cancers among male patients were; colorectal (n = 17, 14.9%) lung and bronchus (n = 16, 14.0%), central nervous system (n = 13, 11.4%), testis (n = 8, 7.0%), and stomach (n = 7, 6.1%). The five most common cancer by site among females were; breast (n = 57, 49.1%) ovary (n = 9, 7.8%), colorectal (n = 8, 6.9%), stomach (n = 5, 4.3%) and central nervous system (CNS) (n = 3, 2.6%) (Table 3). The types of cancer according International Classification of Diseases for Oncology (ICD-O) by sex and age is given in Additional file 1: Table 3a. The stage distribution according to the topography codes are given in Additional file 1: Table 3b.

**Table 3:** 10 most common cancers in adult Syrian refugee patients

Cancer	Male		Female		Total	
	n	%	n	%	n	%
Breast	–	–	57	49.1	57	24.8
Colorectal	17	14.9	8	6.9	25	10.9
Lung and bronchus	16	14.0	1	0.9	17	7.4
Central nervous system	13	11.4	3	2.6	16	7.0
Stomach	7	6.1	5	4.3	12	5.2
Ovary	–	–	9	7.8	9	3.9
Testis	8	7.0	–	–	8	3.5
Pancreas	5	4.4	2	1.7	7	3.0
Bladder	6	5.3	–	–	6	2.6
Larynx	5	4.4	–	–	5	2.2
Others	37	32.5	31	26.7	68	29.6
Total	114	100.0	116	100.0	230	100.0

**Fig. 1:**
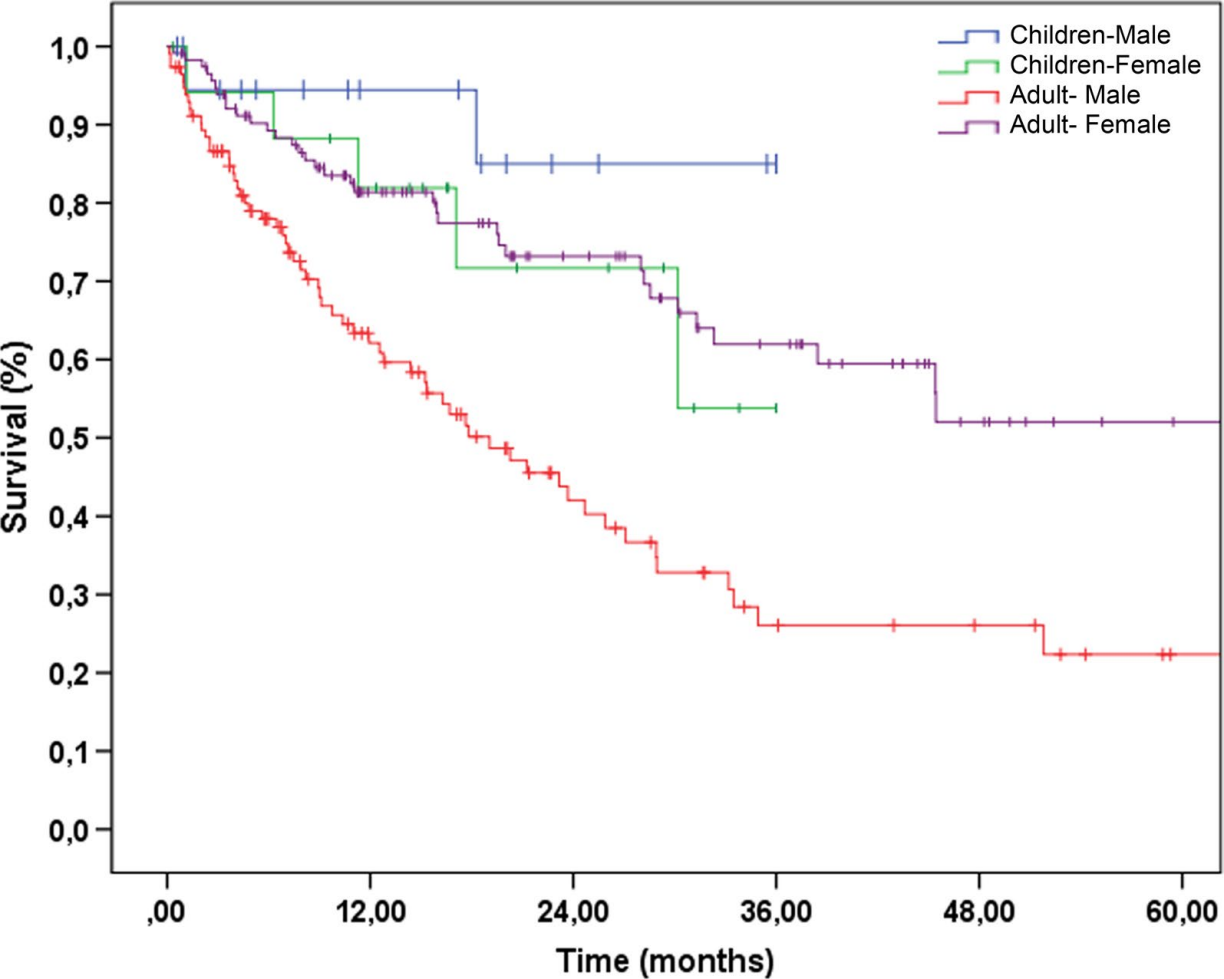
Overall survival of Syrian refugee

The data regarding time to diagnosis from first cancer-related symptom was available in 110 patients and with median 96.5 (IQR = 53–165) days. With regards to the treatment modalities, the majority of patients received more than one type of treatment (Table 4, Additional file 2: Fig. 1). The overall survival probability for the whole group was 44.7% at three years and 37.5% at 5 years. (3 and 5 years survival for males 26.1% and 22.3%; for females 61.9%, 52.0% respectively.) (Fig. 1).The median follow-up time for this cohort was 26.5 months.

**Table 4:** Treatment modalities in Syrian Cancer patients

Type of procedure		Adults	Children
Chemotherapy	Neoadjuvant chemotherapy	29	3
	Adjuvant chemotherapy	100	35
	Palliative	133	1
	Total	262	39
Surgery	Biopsy	131	18
	Curative/complete	112	18
	Curative/partial resection	29	2
	Palliative	13	–
	Other	1*	1**
	Total	286	39
Radiotherapy	Neoadjuvant	3	–
	Adjuvant	63	–
	Therapeutic	17	16
	Palliative	52	–
	Prophylactic radiotherapy	1	–
	Total	136	16
Total		684	94

*LN dissection. details unknown, **Liver Transplant

### Cancer among children

Between August 2014 and December 2020, 38 cases of childhood cancer were identified in the same hospital databases. There were 20 boys and 18 girls and their median age at diagnosis was 5.8 years and 6.0 years respectively. Most (n = 29) had a diagnoses between 2018–2020. The rest were diagnosed between 2014–2017. Except for two cases the cancer was first diagnosed in Turkey. Moreover, 34 (89.5%) patients were living in Turkey during their treatment while three were visiting Turkey just for treatment and returning back to Syria while holding their refugee status (Visiting refugee) (Table 1).

36 (94.7%) patients were newly diagnosed, whereas two patients presented with cancer recurrence. Except for four patients, the rest were diagnosed in the hospitals included in this study. Histopathological examination was the most common method of diagnosis and was applied in 29 (76.3%) patients. Excluding Leukemia cases, among 13 (43.3%) patients the stage of cancer at the time of diagnosis was Advanced (Stage III and IV). Four (10.5%) patients had treatment abandonment (Table 5). Associated genetic abnormalities; WAGR syndrome, Klinefelter syndrome, and ataxia-telangiectasia were found in three children.

**Table 5:** Tumor characteristics in Syrian children with cancer

Characteristics		Male	Female	Total	
		n	n	n	%
Disease status	Newly diagnosed disease	19	17	36	94.7
	Relapsed disease	1	1	2	5.3
Diagnostic method	Histological diagnosis from primary	15	13	28	73.7
	Histological diagnosis from metastasis	1	–	1	2.6
	Cytological diagnosis	4	4	8	21.1
	Clinical examination	–	1	1	2.6
Dissemination of the disease*	Local (Stage I and II)	9	8	17	56.7
	Advanced (Stage III and IV)	7	6	13	43.3
Treatment abandonment	Yes	3	1	4	10.5
	No	17	17	34	89.5
Total		20	18	38	100.0

*Leukemia were not included in staging

When analyzing the whole group, the three most common cancer by ICCC 3 were; Leukemias in eight children (21.1%), Lymphomas and reticuloendothelial neoplasms in eight children (21.1%), and CNS neoplasms in five children (13.2%) (Table 6). The stage distribution according to the cancer classification is given in Additional file 1: Table 4.

**Table 6:** Tumor types in Syrian children with cancer

Tumor types in ICCC3 tumor groups	Male	Female	Total		M/F
	n	n	n	%	
01 Leukemias. myeloproliferative diseases. and myelodysplastic diseases	4	4	8	21.1	1.0
02 Lymphomas and reticuloendothelial neoplasms	5	3	8	21.1	1.7
03 CNS and miscellaneous intracranial and intraspinal neoplasms	2	3	5	13.2	0.7
04 Neuroblastoma and other peripheral nervous cell tumors	1	1	2	5.3	1.0
06 Renal tumors	2	2	4	10.5	1.0
07 Hepatic tumors	–	1	1	2.6	–
08 Malignant bone tumors	–	3	3	7.9	–
09 Soft tissue and other extraosseous sarcomas	3	–	3	7.9	3.0
10 Germ cell tumors. trophoblastic tumors. and neoplasms of gonads	2	–	2	5.3	2.0
11 Other malignant epithelial neoplasms and malignant melanomas	–	1	1	2.6	–
12 Other and unspecified malignant neoplasms	1	–	1	2.6	1.0
Total	20	18	38	100.0	1.1

The data regarding time to diagnosis from first cancer-related symptom was available for 34 patients with a median of 23 days (IQR = 13.5–59). With regards to the treatment modalities, the majority were received more than one modality of treatment. It is worth noting that one patient also underwent liver transplantation (Table 4, Additional file 2: Fig. 2). The two-years overall survival probability was 78.1%, dropping to 69.5% at the end of three years (Two and three years survival for males; 85% and 85%; for females 71.7%, 53.8% respectively.) (Fig. 1). The median follow-up time for all children was 20 months.

## Discussion

Measuring the burden of cancer is vital to understand its impact on the refugee populations as well as to design cost effective, affordable and equitable control and management strategies. Several factors make it difficult to measure the burden of cancer among the refugee population. Availability of the data is one of the biggest challenges as most refugees live in low to low-middle income settings [15] where national statistical systems are either absent, weak or do not include refugees in the publications of national statistical reports due to sensitive political reasons. And even if collected access to scuh data is a challenge. Residential instability, the incomplete identity of refugees, incomplete sharing of data among different stakeholders, the quality and completeness of data are other significant challenges. Hence, the available data most often focus on registered economic migrants in high-income countries [16–18]. At present more than 5 million (UNHCR 2021 n = 5,221,588) Syrian refugees are in Turkey, Lebanon, and Jordan [19] and making this one of largest displaced refugees population in the world. Studies from these countries have focused on the “classical” areas of refugees health. There is limited literature available about cancer and the true burden of cancer is still unmeasured. The most recent systematic review focusing on the impact of armed conflict on cancer among civilians in LMICs reflected the relative paucity of basic epidemiological data including changes to risk factor exposure, behavioral changes, delays to presentation, the availability of timely and affordable complex care, as well as the ability to access care [6].

Konya is the largest province of Turkey by size and currently hosts 121,064 Syrian refugees [11]. The population movement is relatively stable in Konya, in contrast to other cities. Syrian refugees can get the health care only in cities where they are registered to live. Their treatment costs are covered if they are treated in the state or public university hospitals. The referrals from other cities’ government hospitals are permitted for specific pre-authorized cases. The number of refugees who reside in Konya has increased markedly in previous years, growing the cancer burden amongst this population. However, similar to other refugee studies, such as in Jordan, the difference expected and observed number of Syrian cancer patients across the years implies routine under-reporting [20].

One of the previous studies using Turkish Ministry of Health public hospital data for years 2012–2015 reported that the breast, colon, and lung were the most common cancer types observed among adults older than 19 years [21]. Another single-center analysis of 134 adult Syrian cancer cases in the city of Şanlıurfa for years 2015–2017 also reported that breast cancer is the most frequent cancer [22]. The findings of our study were in general in agreement to these studies. Moreover, the frequency of different types of cancers among the refuges was comparable to the host population. The Globocan (global cancer observatory) estimates suggest lung, breast, and colon were the most common cancer types among the host population in Turkey in the year 2020 [23]. Our finding that 40.4% of Syrian refugee patients presented at an advanced stage of their disease was similar to the above mentioned study at Şanlıurfa where 44.8% of all cases presented at an advanced stage. The median age (49.0 years) of adult patients at presentation in the present study is also comparable to the study in Şanlıurfa (47.5 years) [22].

Smoking is one of the major risk factors for various types of cancer. The smoking rate in the present study (30.6%) is similar to Şanlıurfa study (32.8%) [22]. Moreover, the Health Status Survey of Syrian Refugees in Turkey shows that 31.6% of the Syrian refugees smoke a tobacco product daily [24]. The high smoking rate among male population in our study explains why tobacco related tumors are more common in males than females (lung and bronchus, bladder, larynx). The survey results also showed low physical activity, and a diet that does not meet the healthy recommendations is also prevalent among Syrian refugees [24]. Additionally, changes in the socio-cultural, physical, and economic environment due to migration also causes changes in risk for different types of cancers. Considering the high prevalence of these risk factors, host countries must expand their preventive interventions through public health programs and public policy as well as routine cancer care.

To the best of our knowledge, this is the first study that has estimated the median interval between the first cancer symptom and the cancer diagnosis in Syrian refugees. The median diagnostic interval was 96.5 (IQR = 112) days for adult patients. This is a significant delay and reflects the complex and long pathways Syrian refugees need to take, as well as a host of other factors e.g. economic factors that contribute to diagnostic and treatment delays. Earlier research among lung cancer patients in the Eastern Black Sea region of Turkey found that the patients presented on average 30 days (range 2–365) after symptoms with a very wide range [25]. Delay in diagnosis and treatment are independently increase mortality [26]. Strategies addressing delays in diagnosis and treatment for refugees are crucial for downstaging and increasing the proportion of patients who present with curative disease. The cancer control department in Turkey is working closely with primary healthcare physicians to alleviate the situation with various screening guidelines and more rapid cancer care referral pathways. However, the health literacy of Syrians in Turkey is inadequate [27] and many patients ignore or self-managing critical symptoms. It is noteworthy that MoH has established mobile cancer screening units for Syrian refugees which also acts to constructively engage in increasing awareness of cancer symptoms By November 2020 more than 420,000 refugees had been screened through this service [28].

Multimodal combination therapy is a cornerstone of cancer treatment [29]. Our study showed that refugee cancer patients in Turkey have access to all types of modern cancer treatment modalities and they are not, per se, a clinically underserved population. Interruption of ongoing treatment, however, is an important issue in cancer care. There are few cases of treatment abandonment in our population. However, due to the retrospective nature of the study and limited availability of such data in the hospital record the result need to be interpreted with caution as it is not very clear whether the patients truely abdonded their treatments or moved to a different location and were effectively lost to follow up but still being treated.

The overall 5-year survival rate of 37.5% for adult Syrian cancer patients was lower than the host population [23] mostly reflecting delays in presentation and diagnosis.

Two previous studies reported on childhood cancer among Syrian refugees. Kebudi et al. [30], evaluated retrospective data of 212 refugee cancer children from 17 centers in 2015, where leukemia, lymphoma, and brain cancers were the most common cancer types. Yağcı et al. [31], evaluated single-center data of 105 Syrian cancer children in the Adana province where CNS tumors, leukemia, and lymphoma were the most frequent site specific childhood cancers. Though the number of childhood cancer patients in the present is small the frequency of different types of cancer was similar to other studies. The delayed presentation, treatment abondement is an important concern for refugees’ children with cancers. Yağcı et al. [31], found that refugee children had a high frequency of advanced/metastatic disease, and lower treatment compliance compared to host children. We found overall survival for children with cancer at two years as 78%, although due to the small number of cases the interpretation is difficult. However, a previous study reported that overall survival was 55.7% in Syrian cancer children and this rate was significantly lower than host children receiving oncologic treatment [31].

The cost of the cancer care for Syrian refugees is not completely known but the raw estimates based on crude incidence suggest an expenditure of €33·68 million (Turkey €25·18 million; Lebanon €6·40 million; Jordan €2·09 million) [10]. Nevertheless, the financial costs of cancer care are high for both patients and the health system. Syrian refugees in Turkey are in a much better position than many other places as most of the healthcare services are available at freely without discrimination [32]. Moreover, as observed in the current study, to benefit from the health system cancer patient from Syria migrate temporarily to Turkey, obtain refugee status, undergoing cancer treatment, and go back and live in Syria. It is also important to note that the conflict environment has resulted in the disruption of cancer care in Syria and only 23% of functional public hospitals in Syria provide cancer treatments [5]. Thus incidence of cancer may have been over-reported in Syrian refugees living in host countries [20]. This speaks the need to build up capacity, capability and maintain proper computerized patient records in host country systems for externally displayed and also internally displayed refugees back in Syria.

In summary, Cancer is not a neglected disease for the Syrian refugees living in Turkey. This study found that Syrian patients can access cancer care in public and university hospitals. Moreover, the diagnostic and treatment costs for non-communicable diseases including cancer are covered by the government [32]. Nevertheless, delayed diagnosis is an important issue. We found that the median time between the onset of symptoms and diagnosis of cancer is three months for adult patients. As to the retrospective nature of the study, we are unable to identify the reason for the delay. It could be related with migration process itself and other additional factors including financial barriers, the difficulties on access to care due to registration requirement, health literacy, other socio-cultural reasons. Treatment abandonment is also an important factor that limits the survival of cancer. We found that near 10% of adult cancer patients abandoned the cancer treatment. Previous studies highlighted the issue of displacement and interruption of cancer treatment among Syrian refugees [5, 33]. We found a high proportion of lung, larynx, and bladder cancer among men. This could be explained by the high prevalence of smoking among men as compared to women. We also found that the metastatic disease and treatment abandonment were slightly higher in men. However, the limited number of cases makes the comparison difficult. Females also have better survival rates than in males in this study. Based on our study we recommend that the management of cancer should not be ignored at the time of humanitarian crises. Investment on the health system capacity is essential. Having the crisis plan ready for such humanitarian emergencies and developing the new funding models are required. The states of the world must also focus on how to prevent and protect people of the world from the harm of conflict and forced displacement. Research and data collections are also among the major obligations to better understand and proper management the such major crisis.

The results of this study are subject to some limitations. The retrospective nature of the study design is the major limitation. Additionally, the data for this study come from the hospital database, which was not collected primarily for research purposes therefore analysis was limited to the available variables only. Furthermore, due to a large number of missing values for some variables of interest such as smoking and the presence of chronic disease, the conclusion regarding these variables should be interpreted with caution. The absolute incidence or prevalence of cancer cannot be deduced from this data, because the patients diagnosed and/or treated at Konya might come from another city through the referral process, thus it is not possible to determine the precise reference population for this study. The relatively low number of pediatric patients also limits the interpretation of results. Future prospective studies with large sample sizes and better study designs are required to confirm.

## Conclusion

Investment in NCDs including cancer among the refugee and displaced people must be included in national health strategies and cancer control plans. Identifying and responding to the health needs of refugees at the time of sudden and mass influx is challenging for the host countries. It is the responsibility of the international community to collaborate with the host country to address health challenges linked with refugee crisis. The countries bordering the conflict zones should include “emergency crisis plans” for control of NCDs including cancer. Larger, prospective studies are needed to better understand cancer among Syrian refugees and to develop interventions that can be scaled up to reduce late stage diagnosis and provide equitable, affordable and sustainable health care.

## Supplementary Information

**Additional file 1.** Appendix A, Tables containing “Number of cancer diagnosis by year”; “The number of Comorbidities observed among 224 adult Syrian refugee patients”; “Locations of malignancy among refugees by age and sex (M/F)”, “Stage by cancer topography codes" and "Tumor staging in Syrian Children with Cancer”.

**Additional file 2.** Appendix B, Treatment modalities for adult and children Syrian refugee patients.

## Data Availability

The datasets used and/or analyzed during the current study are available from the corresponding author on reasonable request.

## Abbreviations

NCD: Non-communicable disease
CNS: Central nervous system
ICCC-3: Classification of childhood cancer third edition
ICD-10: International classification of diseases, tenth edition
IQR: Interquartile range
SPSS: Statistical package for the social sciences
SEER: Surveillance epidemiology and end results
WAGR syndrome: Wilms’ tumor, aniridia, genitourinary abnormalities, mental retardation syndrome
