# Supplementary material for: Cancer among syrian refugees living in Konya Province, Turkey
Source: Confl Health. 2022 Jan 31;16:3. doi: 10.1186/s13031-022-00434-4 (PMC8805424; doi:10.1186/s13031-022-00434-4)
Supplement: Supplementary file 1 — Additional file 1. Appendix A, Tables containing “Number of cancer diagnosis by year”; “The number of Comorbidities observed among 224 adult Syrian refugee patients”; “Locations of malignancy among refugees by age and sex (M/F)”, “Stage by cancer topography codes" and "Tumor staging in Syrian Children with Cancer”. [file 13031_2022_434_MOESM1_ESM.docx]

**Supplementary Figure 1. Treatment modalities for adult Syrian refugee patients.**


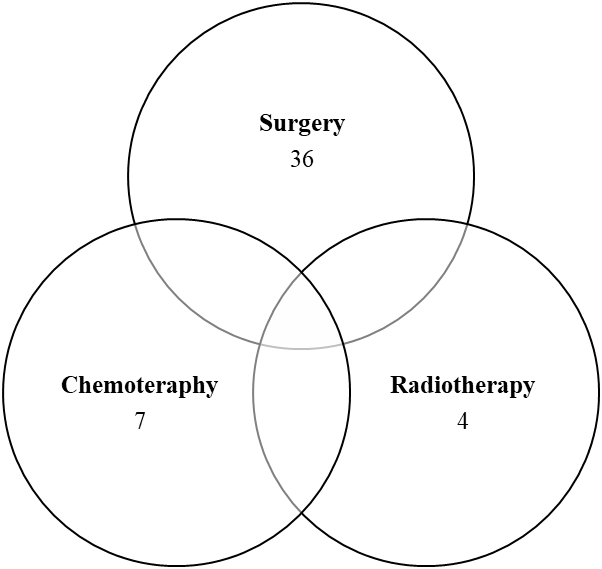


79

22

80

2

**Supplementary Figure 2. Treatment modalities for Syrian Children with Cancer**


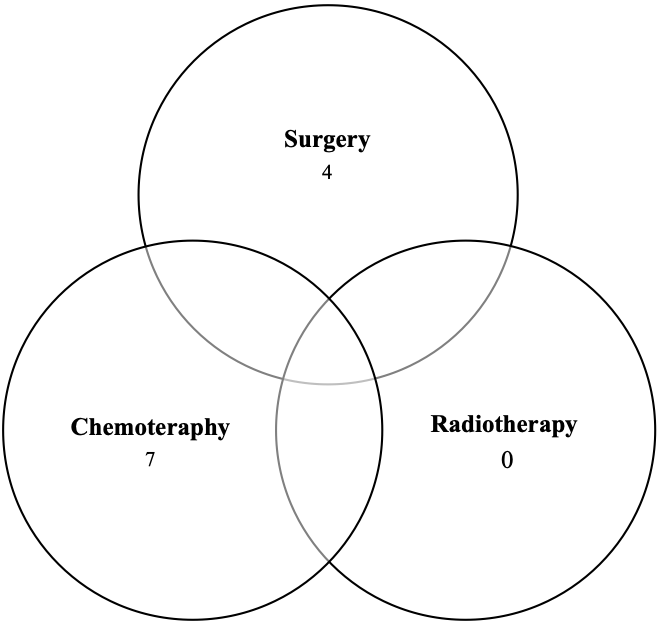


13

2

11

1
