# Supplementary material for: Cancer among syrian refugees living in Konya Province, Turkey
Source: Confl Health. 2022 Jan 31;16:3. doi: 10.1186/s13031-022-00434-4 (PMC8805424; doi:10.1186/s13031-022-00434-4)
Supplement: Supplementary file 2 — Additional file 2. Appendix B, Treatment modalities for adult and children Syrian refugee patients. [file 13031_2022_434_MOESM2_ESM.docx]

**APPENDIX**

**Supplementary Table 1. Number of cancer diagnosis by year**

| **Diagnosis Year** | **n** | **%** |
| --- | --- | --- |
| 2005-2010 | 4 | 1.74 |
| 2011 | 1 | 0.43 |
| 2012 | 3 | 1.30 |
| 2013 | 1 | 0.43 |
| 2014 | 16 | 6.96 |
| 2015 | 13 | 5.65 |
| 2016 | 32 | 13.91 |
| 2017 | 43 | 18.70 |
| 2018 | 39 | 16.96 |
| 2019 | 44 | 19.13 |
| 2020 | 34 | 14.78 |
| **Total** | **230** | **100.00** |

**Supplementary Table 2. The number of Co-morbidities observed among 224 adult Syrian refugee patients***

| **Co-morbidity Type**** | **n** |
| --- | --- |
| Hypertension | 25 |
| Diabetes mellitus | 21 |
| Ischemic heart disease | 14 |
| Chronic renal failure | 3 |
| Goiter | 2 |
| Cerebrovascular | 2 |
| Hepatitis B | 1 |
| Cirrhosis | 1 |
| Chronic hepatitis | 1 |
| Tromboflebitis | 1 |
| No co-morbidity | 177 |

*Data were not available in 6 patients

** A single patient may have more than one co-morbidities.

**Supplementary Table 3a. Locations of malignancy among refugees by age and sex (M/F)**

| **Site** | **Male** | | | **Female** | | | **M/F** | **Total** | | |
| --- | --- | --- | --- | --- | --- | --- | --- | --- | --- | --- |
|  | **n** | **%** | **Mean age** | **n** | **%** | **Mean Age** |  | **n** | **%** | **Mean Age** |
| C00-C14 LIP. ORAL CAVITY AND PHARYNX | 5 | 4.39 | 39.22 | 2 | 1.72 | 60.76 | 5:2=2.5 | 7 | 3.04 | 45.37 |
| C15-C26 DIGESTIVE ORGANS | 35 | 30.7 | 54.54 | 16 | 13.79 | 57.77 | 35:16=2.19 | 51 | 22.17 | 55.56 |
| C30-C39 RESPIRATORY SYSTEM AND INTRATHORACIC ORGANS | 21 | 18.42 | 55.51 | 2 | 1.72 | 72.83 | 21:2=10.5 | 23 | 10 | 57.02 |
| C40-C41 BONES. JOINTS AND ARTICULAR CARTILAGE | 3 | 2.63 | 21.07 | 1 | 0.86 | 17.53 | 3:1=3 | 4 | 1.74 | 20.18 |
| C42 HEMATOPOIETIC AND RETICULOENDOTHELIAL SYSTEMS | 3 | 2.63 | 58.23 | 1 | 0.86 | 52.65 | 3:1=3 | 4 | 1.74 | 56.83 |
| C44 SKIN | 2 | 1.75 | 63.21 | 2 | 1.72 | 55.32 | 2:2=1 | 4 | 1.74 | 59.26 |
| C48 RETROPERITONEUM AND PERITONEUM | - | - | - | 1 | 0.86 | 60.43 | - | 1 | 0.43 | 60.43 |
| C49 CONNECTIVE. SUBCUTANEOUS AND OTHER SOFT TISSUES | 7 | 6.14 | 27.54 | 2 | 1.72 | 34.36 | 7:2=3.5 | 9 | 3.91 | 29.06 |
| C50 BREAST | - | - | - | 57 | 49.14 | 47.03 | - | 57 | 24.78 | 47.03 |
| C51-C58 FEMALE GENITAL ORGANS | - | - | - | 22 | 18.97 | 43.57 | - | 22 | 9.57 | 43.57 |
| C60-C63 MALE GENITAL ORGANS | 12 | 10.53 | 39.35 | - | - | - | - | 12 | 5.22 | 39.35 |
| C64-C68 URINARY TRACT | 7 | 6.14 | 63.93 | 2 | 1.72 | 52.12 | 7:2=3.5 | 9 | 3.91 | 61.31 |
| C69-C72 EYE. BRAIN. AND OTHERS PARTS OF CENTRAL NERVOUS SYSTEM | 15 | 13.16 | 36.55 | 3 | 2.59 | 38.93 | 15:3=5 | 18 | 7.83 | 36.94 |
| C73-C75 THYROID AND OTHER ENDOCRINE GLANDS | 1 | 0.88 | 76.95 | 1 | 0.86 | 57.67 | 1:1=1 | 2 | 0.87 | 67.31 |
| C77 LYMPH NODES | 3 | 2.63 | 34.37 | 3 | 2.59 | 26 | 3:3=1 | 6 | 2.61 | 30.18 |
| C80 UNKNOWN PRIMARY SITE | - | - | - | 1 | 0.86 | 39.01 | - | 1 | 0.43 | 39.01 |
| **Total** | **114** | **100.00** | **48.03** | **116** | **100.00** | **47.73** | **114/116=0.98** | **230** | **100.00** | **47.88** |

**Supplementary Table 3b. Stage by cancer topography codes**

| **Topography** | **Local  (SEER 0-1)** | | **Regional  (SEER 2-5)** | | **Metastatic  (SEER 7)** | | **Unknown (SEER 9)** | | **Total** | |
| --- | --- | --- | --- | --- | --- | --- | --- | --- | --- | --- |
|  | **n** | **%** | **n** | **%** | **n** | **%** | **n** | **%** | **n** | **%** |
| C00-C14 LIP. ORAL CAVITY AND PHARYNX | 1 | 14.3 | 5 | 71.4 | 1 | 14.3 | - | 0.0 | 7 | 100.0 |
| C15-C26 DIGESTIVE ORGANS | - | 0.0 | 24 | 47.1 | 27 | 52.9 | - | 0.0 | 51 | 100.0 |
| C30-C39 RESPIRATORY SYSTEM AND INTRATHORACIC ORGANS | 4 | 17.4 | 3 | 13.0 | 16 | 69.6 | - | 0.0 | 23 | 100.0 |
| C40-C41 BONES. JOINTS AND ARTICULAR CARTILAGE | 1 | 25.0 | 2 | 50.0 | 1 | 25.0 | - | 0.0 | 4 | 100.0 |
| C42 HEMATOPOIETIC AND RETICULOENDOTHELIAL SYSTEMS | - | 0.0 | - | 0.0 | 4 | 100.0 | - | 0.0 | 4 | 100.0 |
| C44 SKIN | 1 | 25.0 | 3 | 75.0 | - | 0.0 | - | 0.0 | 4 | 100.0 |
| C48 RETROPERITONEUM AND PERITONEUM | - | 0.0 | 1 | 100.0 | - | 0.0 | - | 0.0 | 1 | 100.0 |
| C49 CONNECTIVE. SUBCUTANEOUS AND OTHER SOFT TISSUES | 3 | 33.3 | 4 | 44.4 | 2 | 22.2 | - | 0.0 | 9 | 100.0 |
| C50 BREAST | 7 | 12.3 | 30 | 52.6 | 20 | 35.1 | - | 0.0 | 57 | 100.0 |
| C51-C58 FEMALE GENITAL ORGANS | 4 | 18.2 | 8 | 36.4 | 10 | 45.5 | - | 0.0 | 22 | 100.0 |
| C60-C63 MALE GENITAL ORGANS | 5 | 41.7 | 3 | 25.0 | 4 | 33.3 | - | 0.0 | 12 | 100.0 |
| C64-C68 URINARY TRACT | 2 | 22.2 | 4 | 44.4 | 3 | 33.3 | - | 0.0 | 9 | 100.0 |
| C69-C72 EYE. BRAIN. AND OTHERS PARTS OF CENTRAL NERVOUS SYSTEM | 16 | 88.9 | 2 | 11.1 | - | 0.0 | - | 0.0 | 18 | 100.0 |
| C73-C75 THYROID AND OTHER ENDOCRINE GLANDS | - | 0.0 | 1 | 50.0 | 1 | 50.0 | - | 0.0 | 2 | 100.0 |
| C77 LYMPH NODES | - | 0.0 | 2 | 33.3 | 4 | 66.7 | - | 0.0 | 6 | 100.0 |
| C80 UNKNOWN PRIMARY SITE | - | 0.0 | - | 0.0 | - | 0.0 | 1 | 100.0 | 1 | 100.0 |
| **Total** | **44** | **19.1** | **92** | **40.0** | **93** | **40.4** | **1** | **0.4** | **230** | **100.0** |

**Supplementary Table 4. Tumor staging in Syrian Children with Cancer ***

| **Tumor types in ICCC3 tumor groups** | **Local** | **Advanced** | **Total** |
| --- | --- | --- | --- |
|  | **n** | **n** | **n** |
| 02 Lymphomas and reticuloendothelial neoplasms | 2 | 6 | 8 |
| 03 CNS and miscellaneous intracranial and intraspinal neoplasms | 5 | 0 | 5 |
| 04 Neuroblastoma and other peripheral nervous cell tumors | 1 | 1 | 2 |
| 06 Renal tumors | 2 | 2 | 4 |
| 07 Hepatic tumors | 0 | 1 | 1 |
| 08 Malignant bone tumors | 2 | 1 | 3 |
| 09 Soft tissue and other extraosseous sarcomas | 1 | 2 | 3 |
| 10 Germ cell tumors. trophoblastic tumors. and neoplasms of gonads | 2 | 0 | 2 |
| 11 Other malignant epithelial neoplasms and malignant melanomas | 1 | 0 | 1 |
| 12 Other and unspecified malignant neoplasms | 1 | 0 | 1 |
| **Total** | **17** | **12** | **29** |

*Leukemias were not included in staging
